# Comparative studies of four cumin landraces grown in Egypt

**DOI:** 10.1038/s41598-024-57637-3

**Published:** 2024-04-05

**Authors:** Abeer A. Ahmed, Marwa Ghoneim, Mahmoud A. A. Ali, Alia Amer, Aleksandra Głowacka, Mohamed A. A. Ahmed

**Affiliations:** 1 Seed Technology Research Department, Field Crops Research Institute, Agriculture Research Center, Giza, Egypt; 2 Cell Department, Field Crops Research Institute, Agriculture Research Center, Giza, Egypt; 3 Horticultural Department, Faculty of Agriculture, Ain Shams University, Cairo, Egypt; 4 Medicinal and Aromatic Plants Research Department, Horticulture Research Institute, Agricultural Research Center, Giza, Egypt; 5 Department of Plant Cultivation Technology and Commodity Sciences, University of Life Sciences in Lublin, 13 Akademicka Street, 20-950 Lublin, Poland; 6 Plant Production Department (Horticulture - Medicinal and Aromatic Plants), Faculty of Agriculture (Saba Basha), Alexandria University, Alexandria, 21531 Egypt

**Keywords:** Agricultural genetics, Plant sciences, Plant physiology, Plant stress responses, Secondary metabolism

## Abstract

One of the significant aromatic plants applied in food and pharma is cumin. Despite its massive trading in Egypt, there are no comprehensive reports on cumin landraces profile screening. This study aimed to investigate the variation in seeds’ physical and biochemical profiles and genetic diversity as well as assess the efficiency of seeds’ germination under salinity stress. Consequently, during the 2020/2021 growing season, four common cumin seed landraces were gathered from various agro-climatic regions: El Gharbia, El Menia, Assiut, and Qena. Results showed a significant variation in physical profile among the four seeds of landraces. In addition, Assiut had the highest percentage of essential oil at 8.04%, whilst Qena had the largest amount of cumin aldehyde, the primary essential oil component, at 25.19%. Lauric acid was found to be the predominant fatty acid (54.78 to 62.73%). According to ISSR amplification, El Menia presented a negative unique band, whereas other landraces offered a positive band. Additionally, the cumin genotypes were separated into two clusters by the dendrogram, with El Gharbia being located in an entirely separate cluster. There were two sub-clusters within the other cluster: El Menia in one and Assiut and Qena in the other. Moreover, the germination sensitivity to the diverse salinity concentrations (control, 4, 8, 12, and 16 dS/m) findings showed that landraces exhibited varying responses to increased salinity when El Gharbia and El Menia showed a moderate response at four dS/m. Whilst, Qena landraces showed supreme values among other landraces under 12 and 16 dS/m. The majority of the examined features had strong positive associations over a range of salinity levels, according to phenotypic correlation coefficient analysis. To accomplish the aims of sustainable agriculture in Egypt, it would be imperative that the potential breeding program for cumin landraces consider this screening study.

## Introduction

Medicinal and aromatic plants (MAPs) are receiving significant interest in numerous fields. Nowadays, MAPs are used as new sources beneficial to human soundness and with different curative effects^1^. Numerous species have a significant economic value and are used as condiments in food, cosmetics as well as perfumes. Cumin (*Cuminum cyminum* L.), is an annual plant belonging to the Apiaceae family, produces nutritional seeds. These seeds are recognized as separate or paired carpels and have a nine ridges striped pattern and canals^2^. Moreover, cumin contains different elements such as manganese and iron^3^ and has different pharmacological properties such as carminative, anticancer, antioxidant, and stimulant as well assed in perfume, cosmetics, and food industries^4–8^. The origins of cumin are Egypt, the eastern Mediterranean, and Turkistan. Recently, it has been widely distributed in China, Iran, Morocco, India, Japan, southern Russia, Turkey, Indonesia, and Algeria^9–11^. Cumin seed’s flavor is due to the presence of volatile oil, which differs depending on its origin and variety^12,13^. The chemical composition of cumin oil shows variations that may be attributed to the difference in the geographical localities, climatic situations, or varieties^12,14–16^. Depending on the growing region and the processing practices, the percentage of the essential oil in cumin seeds ranges from 2 to 5%^17–19^. Cumin aldehyde, Y-terpinene, 7-alpha cymene, and -pinene have been identified as the primary components of essential oil in research undertaken in various countries^6,20^. Fixed oils can also be found in cumin seeds with a proportion ranging from 10 to 20%, when the primary components being 60% oleic acid and 30% linoleic acid^17,21^.

In Egypt, cumin is cultivated during the winter season, covering approximately 1496.5 ha (≈ 3563 feddan) with a total production of about 2036 Tons (Source: Agriculture Economic sector statistics “AESS”2020/2021, Available at: https://www.agri.gov.eg/library/25)^22^. El Menia represents the highest governorate in cumin production at 49%, while El Gharbia produces 29%, then Assiut and Qena governorates at 2% and 0.24%, respectively. Egypt exports most of its cumin seeds to the United States, India, and Indonesia. (https://www.volza.com/exports-egypt/egypt-export-data-of-cumin+seed). The Egyptian variety (Baladi) is distinguished by a high value due to the high percentage of essential oil in it (up to more than 7%). This is because the agro-climatic conditions in Egypt are ideal for growing the premium cumin seed that the international market demands. Although cumin is used extensively in the chemical, pharmaceutical, and health sectors^23^, no studies have been done in Egypt on the breeding and production of cumin varieties that can withstand salinity. Moreover, to gain a thorough understanding of the plant’s characteristics, knowing about genetic diversity and its morphological and biochemical characteristics as well as its growth behavior under salinity conditions is crucial for creating screening selection criteria in plant breeding initiatives^8,24–27^. Cumin has been reported to have a high degree of phytochemical diversity^25,28^, morphological diversity^26,29^, and genetic diversity^28,30,31^.

Furthermore, DNA molecular markers are widely used for plant genetic variation assessment for several advantages e.g. neutral, not related to age and tissue type, not affected by environmental conditions, feasibility, lower costs, and are more informative than morphological markers^32^. In this context, it is considered a reliable method for detecting polymorphism at the level of DNA and could be helpful in genetic relationships within and among species and ecotypes^33^. Several researchers have preferred the Inter simple sequence repeat (ISSR) for marker due to its simplicity, quickness, lower cost, and high reproducibility^34,35^. In addition, ISSR markers are characterized by rapidly revealing high polymorphic fingerprints and have been used frequently to determine the genetic diversity among 18 Iranian black cumin populations^36^. In addition, it was stated that RAPD, SCoT, CCMP, ISSR, and SSR could be used in cumin genetic diversity^37–39^. On the other hand, understanding genetic diversity is necessary to plan conservation and breeding programs for native populations^40,41^.

On the other hand, the seed germination process is the critical stage for the crops’ successful cultivation^42^. It is controlled by several abiotic stresses, including salinity^43,44^. This is due to inadequate irrigation and drainage management mentioned by Ref.^45^. Salt stress is the soluble salt accumulation around the root of a plant or in the deep of plowing to the range that damages the growth of the plant^46^. Under salinity stress, plants’ growth may be decreased with the changes in osmotic potential through little water potential in the root surroundings or through the ion effects^47^. Germination is the plant growth stage severely inhibited by salinity increasing, as mentioned by Sosa et al.^48^. When the germination data are limited and inconsistent for cumin, as reported by Ref.^49^, the cumin plant is considered relatively salt-resistant during adult growth and reproductive stages^19,50^. Moreover, Zidan et al.^51^ found that cumin germination was significantly reduced under high salinity concentrations. Otherwise, Refs.^52,53^ stated that salinity lower than 50 mmol doesn’t affect the cumin vegetative characteristics.

Hence, the present landraces in this study are the leading landraces grown in different geographical areas in Egypt. Therefore, understanding the physical and biochemical profiles and genetic variation in these landraces much crucial for research. In this context, the objectives of the present study were to (1) assess the physical, biochemical, and genetic variation (2) to explore the effect of different salinity stress levels at germination stages, (3) to identify the phenotypic correlation coefficients of quantitative traits among cumin landraces under different salinity levels.

## Results

### Physical properties of cumin seeds

The seed’s dimension, bulk density, and mass of 1000 seeds are listed in Table 1. The results achieved from variance analysis revealed that a significant difference was observed in seed width and weight of 1000 seeds. While no significant differences were seen between seed lengths. The cumin seeds from El Menia governorate showed the highest width being 0.235 mm, followed by cumin seeds from Assiut, then Qena and El Gharbia landraces by 0.206, 0.174, and 0.135 mm, respectively. Qena landrace presented the maximum value of 1000 seeds weight by 0.247 mg, while the minimum value was achieved by El Menia landrace. The seed’s length ranged between 0.638 and 0.672 mm. As for seed bulk density, Table 1 indicated that the highest value being of 1250 kg m^−3^ for the Assiut landrace, while the lowest was 810 kg m^−3^ for the El Gharbia landrace when no significant differences between El Menia and Qena landraces.

**Table 1:** Physical characteristics of cumin landraces.

Landraces	Width (mm)	Length (mm)	Bulk density (kg m^−3^)	1000-seed (mg)
El Gharbia	0.135b	0.638a	810c	0.227ab
El Menia	0.235a	0.647a	1000b	0.207b
Assiut	0.206a	0.669a	1250a	0.213ab
Qena	0.174b	0.672a	1040b	0.247a
F test	*	ns	*	*
LSD	0.05	0.04	126.3	0.04
CV%	7.76	3.4	7.82	8.83

Significant differences are denoted by asterisks: *P ≤ 0.05.

### Phytochemical compounds

#### Essential oil% and GC–MS analysis

The percentage and chemical compositions of the essential oils of cumin seeds from El Gharbia, El Menia, Assiut, and Qena are presented in Table 2. Significant effects on essential oil% were detected regarding the different landraces. The data in Table 2 indicated that the Assiut landrace had the maximum percentage of essential oil (8.04%). While El Gharbia landrace had a minimum value of oil percentage of 5.55%. GC/MS analyses of the isolated essential oil revealed 33 compounds (Table 2). Five components were considered as predominant: β-Pinene (9.59–10.21%), p-Cymene (6.11–8.31%), γ-Terpinene (13.56–14.14%), Cumin aldehyde (22.71–25.19%), γ-Terpinen-7-al (18.27–21.13%). Moreover, α-Terpinene and Phellandral are found in all landraces while absent in the essential oil of Qena landrace. cis-β-Terpineol, l-Pinocarveol, and Carvacrol have been detected only in the essential oil of El Menia landrace. As can be observed, cis-α-Bergamotene, Corymbolone, and 4a, 7, 7, 10a Tetra methyl dodecahydro benzo [f] chromen-3-ol were observed only in the essential oil of the Assuit landrace. Individually, cuminic acid was identified only in the essential oil of the Qena landrace.

**Table 2:** Essential oil % and its composition of the cumin landraces.

No	Landraces	El Gharbia	El Menia	Assiut	Qena
	Essential oil % (v/w)	5.55d	6.03c	8.04a	6.82b
	Constituents	Relative peak area, %			
1	β-Thujene	0.65	0.65	0.62	0.59
2	α-Pinene	1.53	1.50	1.46	1.46
3	Sabinen	1.57	1.64	1.68	1.55
4	β-Pinene	9.59	9.94	10.21	10.13
5	β-Myrcene	1.07	1.17	1.32	1.11
6	α-Phellandrene	0.80	0.81	1.20	0.68
7	α-Terpinene	0.24	0.23	0.27	–
8	p-Cymene	7.53	7.19	6.11	8.31
9	d-Limonene	1.51	1.32	1.27	1.03
10	Eucalyptol	0.27	0.26	0.28	0.25
11	γ-Terpinene	13.79	13.74	13.56	14.14
12	cis-β-Terpineol	–	0.27	–	–
13	l-Pinocarveol	–	0.20	–	–
14	Terpinen-4-ol	0.29	0.28	0.30	0.31
15	3-p-Menthen-7-al	3.32	2.39	3.44	2.11
16	Estragole	1.76	2.22	0.48	1.33
17	Cumin aldehyde	24.82	22.71	24.30	25.19
18	( −)-Carvone	1.95	2.28	–	–
19	Phellandral	0.26	0.20	0.24	–
20	α-Terpinen-7-al	4.80	5.10	4.64	5.11
21	γ-Terpinen-7-al	18.27	21.13	20.65	19.86
22	Carvacrol	–	0.22	–	–
23	p-Mentha-1,4-dien-7-ol	0.85	0.82	1.08	0.79
24	β-Gurjunene	0.74	0.66	0.99	0.69
25	β-Caryophyllene	0.54	0.43	0.77	0.95
26	Cuminic acid	–	–	–	1.24
27	*cis*-α-Bergamotene	–	–	0.23	–
28	(E)-β-Famesene	0.71	0.65	0.96	0.76
29	β-Copaene	0.41	0.38	0.57	0.39
30	4-Epi-α-acoradiene	0.41	0.31	0.44	0.44
31	Carotol	1.70	1.31	2.41	1.58
32	Corymbolone	–	–	0.25	–
33	4a,7,7,10a Tetra methyl dodecahydro benzo [f] chromen-3-ol	–	–	0.27	–

#### Essential oil fatty acids profile

**Figure 1:**
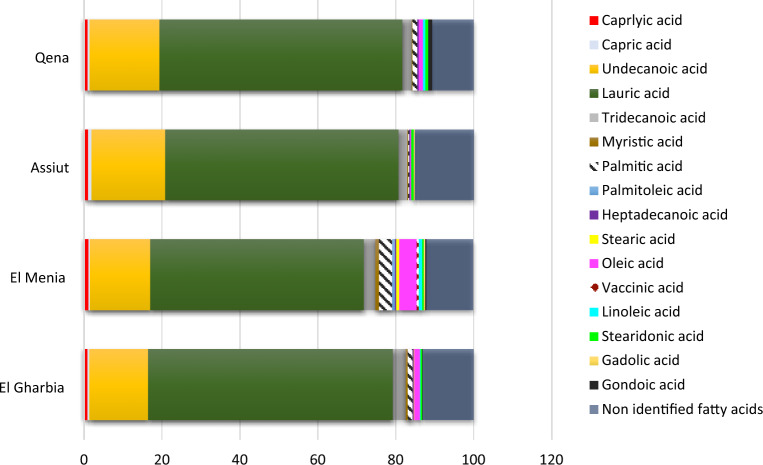
Fatty acid composition of cumin essential oil of the 4 cumin landraces.

Based on GC–MS results, where it spotted the presence of fatty acids and since little is documented about the fatty acids profile of volatile oil^54^, it was of interest to detect the fatty acids profile of cumin essential oil for the first time. There was a significant difference in the landraces on the fatty acids (%) (Fig. 1). The major fatty acid was Lauric acid ranged between 54.78 and 62.73%, where no significant differences were observed in Lauric acid content between El Gharbia and Qena landrace which showed the highest percentage (62.73 and 62.31% respectively). In contrast, El Menia presented the lowest value (54.78%) of lauric acid in the essential oil. The highest supreme value from undecanoic acid (18.93%) was attained from the Assiut landrace. Furthermore, El Menia landrace offered the highest amount of Palmitic acid and Oleic acid (3.29% and 4.39%, respectively.). Meanwhile, palmitic and vaccinic acids have been observed in the El Menia landrace with percentages of 0.77 and 0.55%, respectively, compared to other landraces. In addition, gondoic acid is not perceived in the Assiut landrace compared to other landraces.

### Molecular analysis

#### ISSR amplification

**Figure 2:**
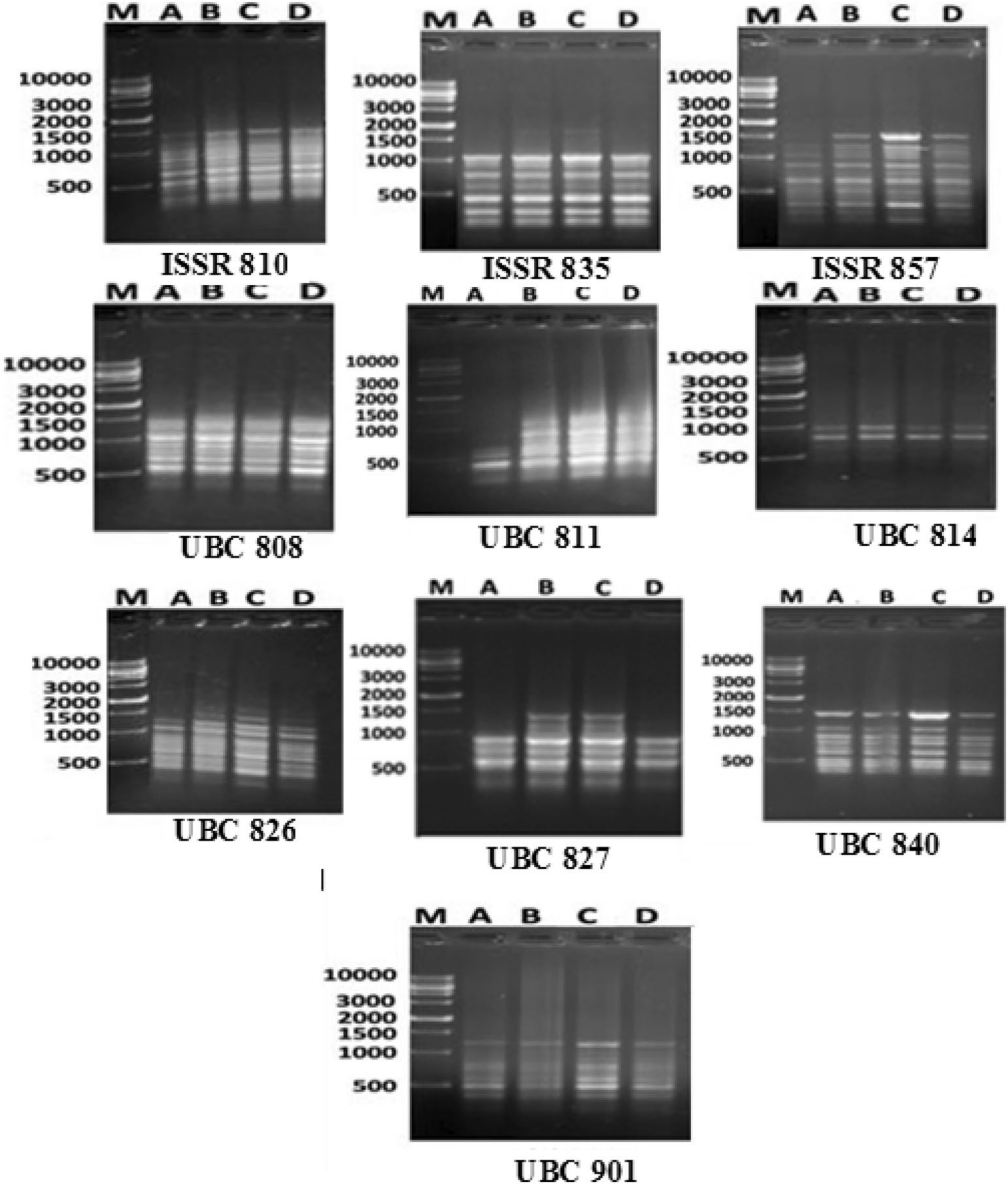
ISSR amplification profile obtained from the 4 cumin landraces [DNA size marker 1 ng/5uL]. M = 10 Kb plus DNA ladder, A: El Menia; B: El Gharbia; C: Assiut; D: Qena.

ISSR products (Fig. 2) and analysis involved 10 primers of the ISSR 97 bands were produced, 72 monomorphic bands, and 25 polymorphic bands (Table 1). Primers no. ISSR 810 and ISSR 857 produced the highest number of bands (13 bands) with 15.4 polymorphism, followed by the primers no. ISSR 7, ISSR 8, and UBC 826 have 11 bands with polymorphisms of 18.2%, 63.6%, and 36.4% respectively. The lowest number of bands was obtained from primer no. UBC 814, which had four bands with a polymorphism of 50% (Table 3). ISSR marker produced 16 unique bands 4 positive and 12 negative; primer no. ISSR 8 had the highest unique bands (5), positive, unique bands (650 pb, 700 pb, 900 pb, 1000 pb, and 1100 pb) characterized by El Menia genotype. Also, El Menia genotype was characterized by negative unique bands (130pb). In addition, El Gharbia genotype had five unique bands, positive unique bands at 800, 1400, and 1000 pb, and negative unique bands at 650 and 500 pb. Moreover, ISSR primers divide genotypes with dendrogram into two clusters. The first had El Gharbia separated from the El Menia, which is found in the next cluster separated from Assiut and Qena, which were close to each other. The highest distance similarity was obtained between El Gharbia and El Menia at 0.218, and the lowest distance between Assiut and Qena. On the other hand, the lowest value for heterozygosity index (H), polymorphism information content (PIC), the arithmetic mean of H (H.av), marker index (MI), Discriminating power (D), and resolving power (RP) was obtained with primer no. ISSR 835 (Table 5); also primer no. ISSR 810 had a low value for all parameters. While primer no. UBC 840 had the highest value for the parameters H (0.5), PIC (0.412), H.av (0.5), MI (0.5), and the lowest value for effective multiplex ratio (E), and D were 1.0 and 0.04, respectively, followed by primer no. UBC 808 had high values for the parameters, H (0.459), PIC (0.407), H.av (0.459), and MI (0.459), while E also (1.0).

**Table 3:** ISSR amplification in 4 Cumin genotypes.

Primers	FR	TB	P	MB	P%	UB	ve + UB	ve- UB	G	H	PIC	E	H.av	MI	D	RP
ISSR-810	200–1400	13	2	11	15.4	1		1350	2	0.097	0.09	12.3	0.00	0.031	0.10	1.33
ISSR-835	200–1200	8	0	8	0.0	0	0	0	0	0.000	0.00	8.00	0.00	0.000	0.00	0.00
ISSR-857	200–1600	13	2	11	15.4	2	800	650	3	0.142	0.13	12.0	0.00	0.04	0.15	1.33
UBC-808	200–1300	11	2	9	18.2	1	0	130	1	0.459	0.41	1.00	0.46	0.46	0.31	
UBC-811	200–1200	11	7	4	63.6	5	0	650, 700, 900, 1000, 1100	1	0.25	0.23	1.00	0.25	0.25	0.11	
UBC-814	300–800	4	2	2	50.0	1	0	500	3	0.38	0.31	3.00	0.03	0.09	0.46	1.33
UBC-826	200–1300	11	4	7	36.4	2	900	0		0.26	0.22	9.33	0.01	0.07	0.28	2.67
UBC-827	200–1400	8	3	5	37.5	2	0	1000, 1200	4	0.22	0.2	7.00	0.01	0.06	0.24	2.00
UBC-840	300–1450	10	1	9	10.0	1	1400	0	3	0.50	0.41	1.00	0.50	0.50	0.04	
UBC-901	400–1200	8	2	6	25.0	1	1000	0	3	0.28	0.24	6.67	0.01	0.08	0.31	1.33
Total		97	25	72	25.8	16				2.58	2.24	61.3	1.27	1.58	2	10
Mean		9.7	2.5	7.2	2.3	1.6				0.26	0.224	6.13	0.13	0.16	0.2	1

*FR* Fragment range, *MB* monomorphic band, *TB* total bands, *P* polymorphism band, *P%* polymorphism percentage, *UB* Unique bands, + *ve* UB positive unique bands, − *ve* UB negative unique bands, *G* Genotype, *H* heterozygocity index, *PIC* polymorphism information content, *E* effective multiplex ratio, *H.av* arithmetic mean of H, *D* Discriminating power, *MI* marker index, RP resolving power.

#### Principle component analysis

**Figure 3:**
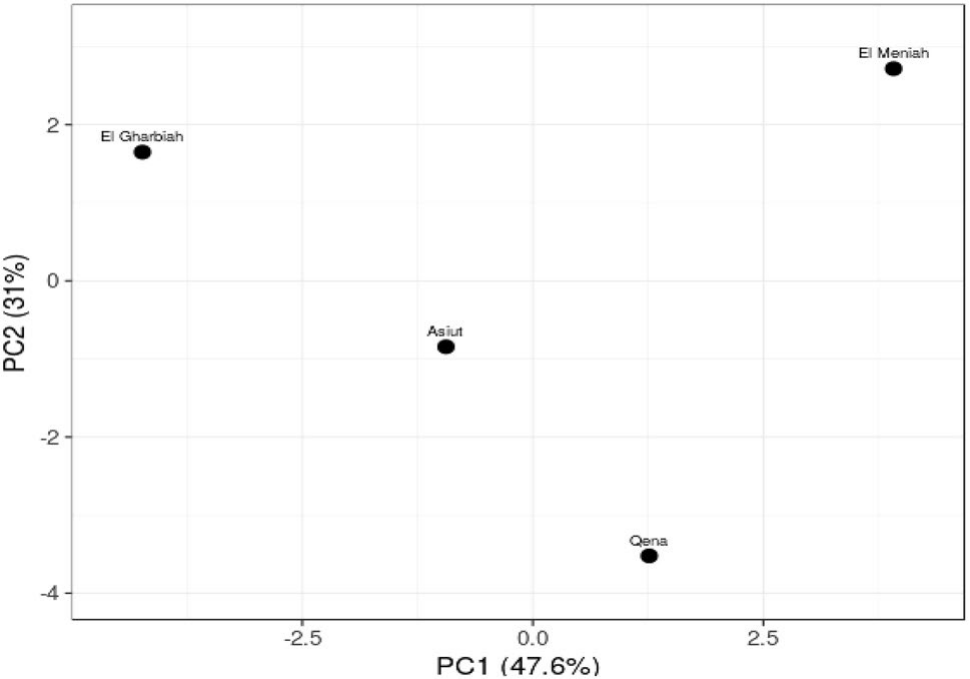
Principle component analysis (PCA) scatter diagram for the 4 cumin landraces.

The PCA scatter diagram (Fig. 3) illustrates the genetic diversity of the 4 cumin genotypes based on their analysis of ISSR marker polymorphism and by blotting the first two principal components using PAST software. The PCA analysis classified the genotypes into four groups, each of which was distinct from the others. El Gharbia and El Menia were detected on the top quadrat of PCA separately but in the same direction. While Assuit and Qena were approximately nearby.

#### Multivariate heat map

**Figure 4:**
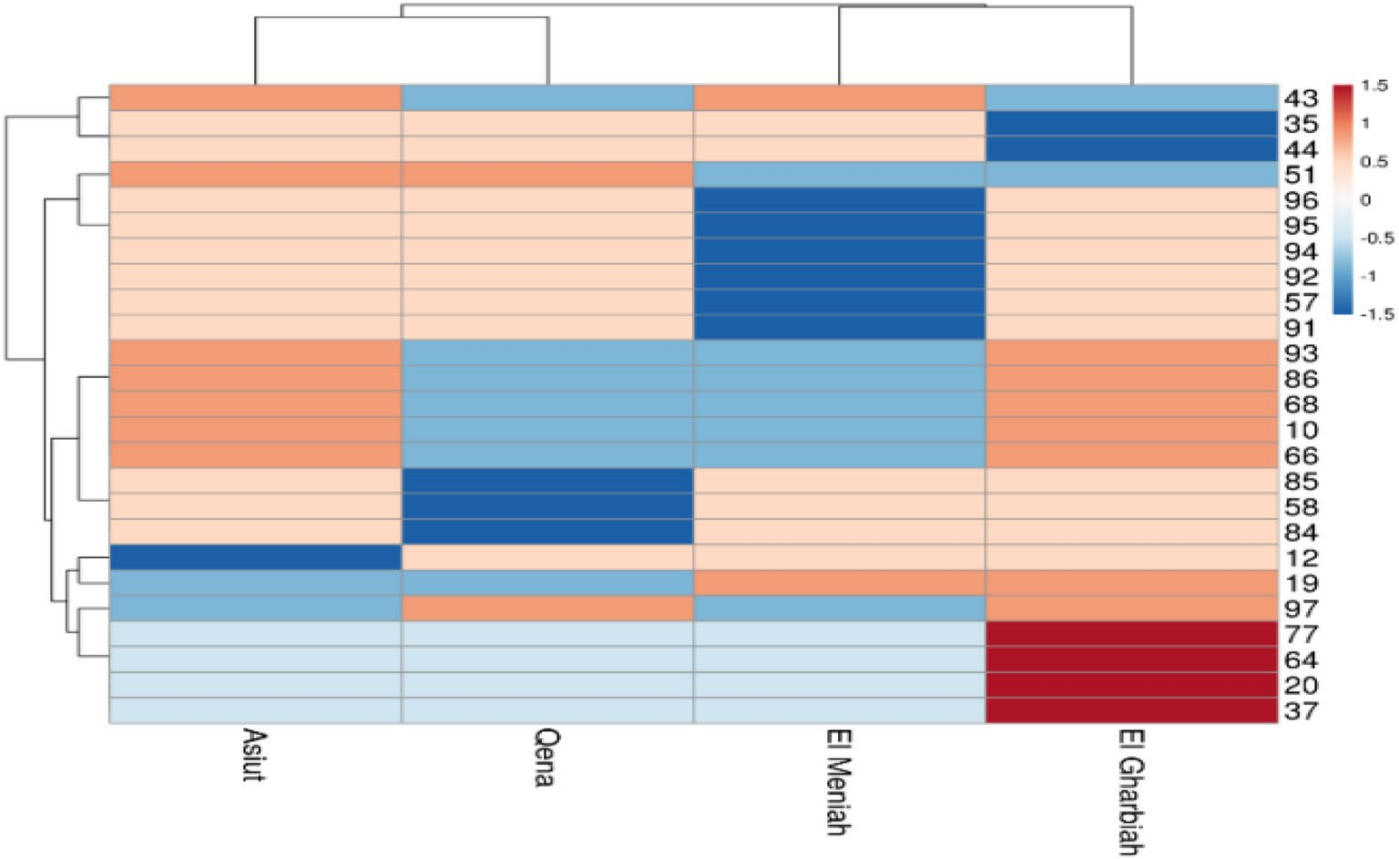
Multivariate heatmap for the 4 cumin landraces based on 10 ISSR primers using the ClustVis heatmap module—a web-based tool for multivariate data clustering and visualizing.

The heat map (Fig. 4) illustrates the genetic diversity of four cumin genotypes based on the 10 ISSR markers constructed using the module of a heatmap of R software. The map indicated that the occupations were distributed into two clusters. The first cluster contains two genotypes, Assiut and Qena, and the second one contains El Menia and El Gharbia genotypes. There is a similarity between the PCA distributions, the heat map distributions, and the dendrogram distributions in the different distributions. In PCA, the genotypes El Menia and El Gharbia were found in the top quarters, and Assiut and Qena were found in the low quarter and close to each other. For the dendrogram, Assiut and Qena were found in the same cluster, and El Menia and El Gharbia were found in separate clusters but near each other.

#### Dendrogram and genetic distances

**Figure 5:**
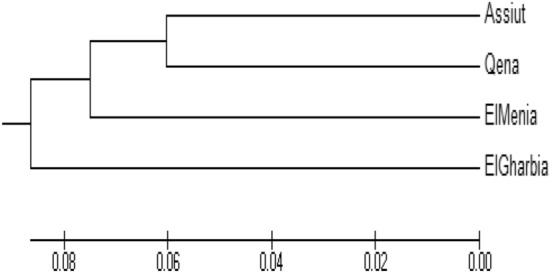
Cluster tree demonstrating the genetic distance, based on the 10 ISSR primers analysis for 4 Egyptian Cumin genotypes using the Euclidean distance and the UPGMA algorithm in the PAST software.

As shown in Table 4 and Fig. 5, the dendrogram divided the cumin genotypes into two clusters: El Gharbia, which was found in the separated cluster from Assiut, Qena, and El Menia. The second cluster had two sub-clusters: one had El Menia, and the second had two genotypes Assiut and Qena. The genetic distances between Assiut and Qena and El Gharbia were 0.12 lower than between El Menia and El Gharbia 0.22 and El Gharbia and Qena 0.2.

**Table 4:** Genetic dissimilarity among four Cumin genotypes based on ISSR banding pattern.

	El Menia	Assiut	El Gharbia	Qena
El Menia				
Assiut	0.16			
El Gharbia	0.22	0.12		
Qena	0.14	0.12	0.2

#### Effect of salinity on seed germination and seedling development

Overall findings revealed that each trait associated with germination and seedling growth was suffered from salt stress. It is worth noting that landraces exposed varying responses to increased salinity.

### Germination characteristics

**Figure 6:**
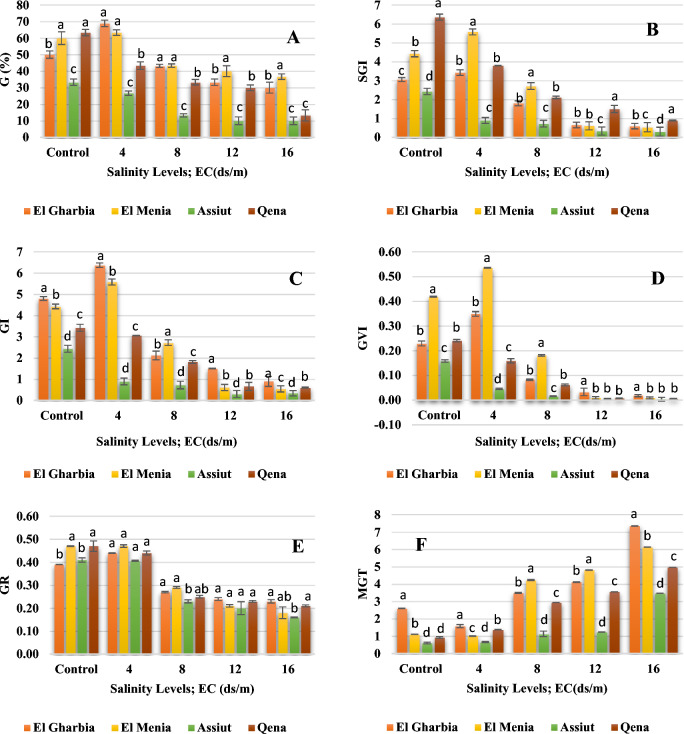
Seed germination percentage (**A**) speed germination index, (**B**) germination index, (**C**) germination vigor index, (**D**) germination rate, (**E**) and mean germination time, (**F**) of cumin seeds treated with different salinity levels. Different letters above bars indicate significantly different means according to the Duncan test (α = 5%) within the same salinity treatment.

**Figure 7:**
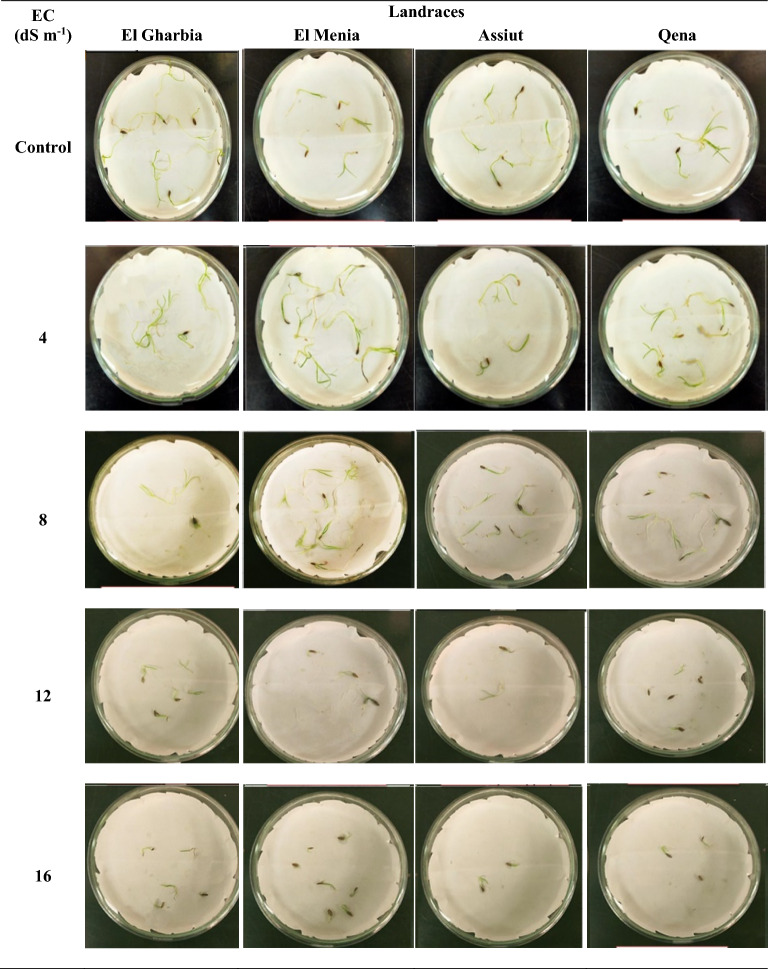
Seed germination of cumin landraces at the different salinity levels.

Data in Figs. 6 and 7 indicated significant differences at (p < 0.05) between all treatments for all seed germination characters. A reduction in all germination characters with increasing the salinity levels from 4 to 16 ds/m was observed. The highest values of G% were observed on El Gharbia and El Menia landraces, while the Assiut landrace recorded the lowest percentage under all salinity levels (Fig. 6A). Compared to other landraces, Qena landrace had the superior value of SGI (1.50 and 0.89) under 12 and 16 ds/m respectively, while El Menia landrace gave the maximum value of SGI (5.57 and 2.71) under 4 and 8 ds/m respectively, as represented in Fig. 6B. Moreover, GI is demonstrated in Fig. 6C; data indicated that El Gharbia landrace reached the maximum GI (6.37) under 4 ds/m compared to the control. Moreover, El Menia landrace recorded 0.54 GVI under 4 ds/m, representing the greatest amount compared to control (Fig. 6D). Meanwhile, Fig. 6E illustrates the GR of the landraces under the salinity levels. It indicates no significant differences between landraces under all salinity levels were observed. On the other hand, a significant increase in MGT was found with increasing salinity levels up to 16 ds/m for all landraces, where El Gharbia showed the supreme amount (7.35), as seen in (Fig. 6F).

### Seedling characteristics

**Figure 8:**
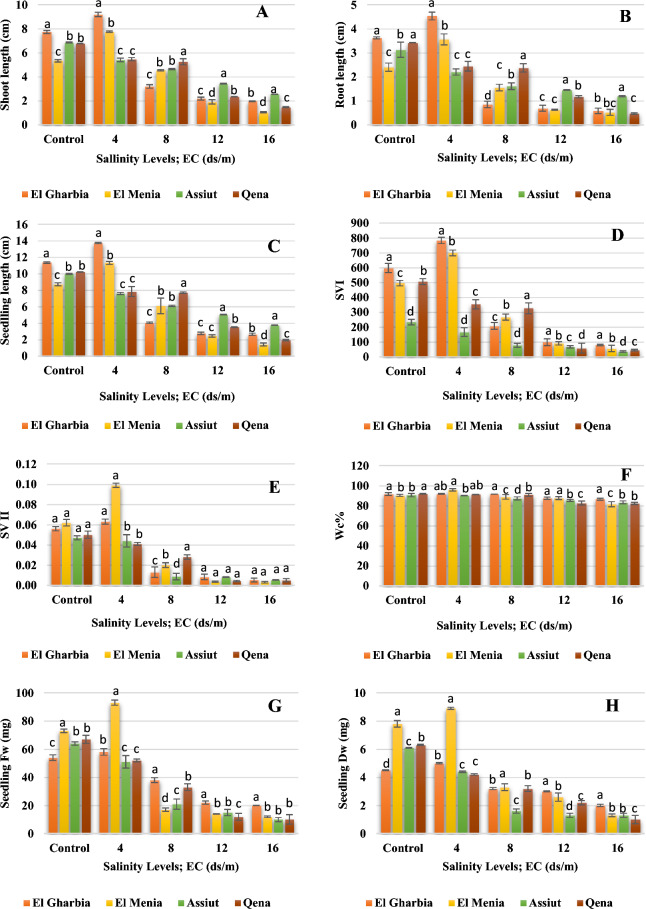
Shoot length (**A**), root length (**B**), seedling length (**C**), SVI (**D**), SVII (**E**), water content (**F**), seedling fresh weight (**G**) and seedling dry weight (**H**) of cumin seeds treated with different salinity levels. Different letters above bars indicate significantly different means according to the Duncan test (α = 5%) within the same salinity treatment.

Figure 8 illustrates the frequency distribution bars of seedling characters of the cumin landraces at different salinity levels. At 4 ds/m of EC, El Menia landraces had the supreme increase by 45.40%, 48.33%, 29.55%, 27.40%, 14.10%, 41.14%, 59.68%, and 6.07% for Shoot length, root length, seedling length, fresh weight, dry weight SV I, SV II, and Water content, respectively compared to control. Moreover, El Gharbia landrace increased by 18.89%, 25.14%, 20.88%, 7.41%, 11.11%, 30.93%, 12.50%, and 0.15% for Shoot length, root length, seedling length, fresh weight, dry weight SV I, SV II, and water content, respectively in comparison with control. On the other hand, Qena landrace under 8 ds/m showed the highest values of shoot length (5.26 cm), root length (2.37 cm), seedling length (7.70 cm), SV I (326), and SV II (0.028).

### Phenotypic correlation coefficients

The evaluation of associations among several characters showed that some characters are positively correlated, while others are negatively correlated. This means it is considered an indicator for increasing or improving a specific character that will have a positive or negative influence on the other characters (Table 5). At all salinity treatments, phenotypic correlation showed that the germination vigor index had a high and positive significant correlation obtained with GI. GR also significantly correlated with seedling dry weight. In addition, seedling length and root length are highly correlated with shoot length. On the other hand, at the control, a highly negative correlation was between shoot and seedling length, with each seedling dry weight by 0.98 and 0.98 and germination rate by 0.78 and 0.72, respectively, root length with seedling dry weight by 0.91, In addition, under 12 ds/m, the high and negative significant correlation achieved between G % and shoot length (0.99); root length (0.88); seedling length (0.98), as well as shoot and seedling length, recorded − 0.91 and − 0.94 with seedling dry weight.

**Table 5:** Phenotypic correlation coefficients of quantitative traits.

Studied characters	Salinity EC, ds/m	Shoot length	Root length	Seedling length	Seedling dry weight	GR	WC%	GI	GVI
G%	Control	− 0.38	− 0.16	− 0.29	0.35	0.74**	− 0.81**	0.25	− 0.03
	4	0.92**	0.94**	0.93**	0.54*	0.78**	0.46*	0.85**	0.70**
	8	− 0.42*	− 0.31	− 0.21	0.95**	0.91**	0.44*	0.96*	0.73**
	12	− 0.99**	− 0.88**	− 0.98**	0.89**	0.52*	0.65*	0.51*	0.41*
	16	− 0.63*	0.99**	− 0.63*	0.49*	0.29	0.08	0.52*	0.64*
Shoot length	Control		0.96**	0.99**	− 0.98**	− 0.78**	0.55*	0.30	0.62*
	4		0.96**	0.99**	0.43*	0.53*	0.33	0.61*	0.46*
	8		0.96**	0.98**	− 0.17	− 0.35	− 0.27	− 0.23	− 0.23
	12		0.91**	0.98**	− 0.91**	− 0.56*	− 0.69*	− 0.54*	− 0.44*
	16		0.51*	0.96**	0.28	− 0.23	0.30	− 0.23	− 0.08
Root length	Control			0.98*	− 0.91**	− 0.57*	0.48*	0.20	0.50*
	4			0.99**	0.37	0.53*	0.42*	0.61*	0.44*
	8			0.951**	− 0.02	− 0.35	0.01	− 0.18	− 0.14
	12			0.83**	− 0.78**	− 0.70**	− 0.66*	− 0.48*	− 0.33
	16			0.52*	− 0.62*	− 0.35	− 0.21	− 0.60*	− 0.74**
Seedling length	Control				− 0.98**	− 0.72**	0.49	0.32	0.63*
	4				0.41*	0.52*	0.35	0.60*	0.45*
	8				0.03	− 0.15	− 0.22	− 0.02	0.13
	12				− 0.94**	− 0.55*	− 0.73	− 0.61*	− 0.53*
	16				0.23	− 0.32	0.21	− 0.31	− 0.14
Seedling dry weight	Control					0.83**	− 0.43*	− 0.46*	− 0.76**
	4					0.81**	− 0.40	0.78**	0.90**
	8					0.80**	0.55*	0.92**	0.65*
	12					0.75**	0.91**	0.84**	0.78**
	16					0.51*	0.76**	0.71**	0.91**
GR	Control						− 0.53*	− 0.40*	− 0.68*
	4						0.18	0.99**	0.98**
	8						0.04	0.97**	0.93**
	12						0.92**	0.87**	0.79**
	16						0.84**	0.95**	0.77**
WC%	Control							− 0.54*	− 0.19
	4							0.26	− 0.01
	8							0.20	− 0.27
	12							0.97**	0.98**
	16							0.85**	0.81**
GI	Control								0.93**
	4								0.96**
	8								0.89**
	12								0.99**
	16								0.93**

* and ** denote significance at 0.05 and 0.01 levels of probability, respectively.

## Discussion

The current investigation demonstrated that the agroecological zone has a more significant effect on the quality of cumin. The observed variation in physical cumin seeds collected from the agroecological zone of Egypt is not too significant due to varietal similarity. Liu et al.^55^ revealed that the seed morphology and the weight of 1000 seeds were positively correlated with longitude and latitude as well as annual average temperature. Additionally, biochemical performances varied significantly between the cumin landraces (Tables 1, 2, Fig. 1). This variability depends on several factors, including geographical location, climate, and genetics. As mentioned before by Refs.^56–59^ on cumin, they reported that the essential oil components depend upon several internal and external factors that affect the plants, including genetic characteristics, ecological status, and agricultural practices. In addition, Refs.^13–61^ recorded that the chemical constituents of cumin oil showed variations that attributed to the varieties or geographical localities differences. These results are in agreement with Ref.^62^ on *Thymus algeriensis*, Ref.^63^ on *Pistacia lentiscus*, Ref.^64^ on *Marrubium vulgare*, and Ref.^65^ on *Coriandrum sativum.*

Furthermore, the relationship between genetic differentiation and ecological factors needs to be explored due to the little information available about the environmental impact on different cumin accessions^39^. Different markers have been recorded for genetic diversity analysis in cumin and other species belonging to the Apiaceae family. Until now, the genetic relationships between cumin ecotypes were examined in terms of agro-morphological characteristics^37,66^ as well as molecular polymorphism using markers such as SSR, RAPD, CCMP, and SCoT^27,67,68^. In addition, Pezhaman et al.^69^ noticed 86% polymorphism after utilizing 38 RAPD primers among 20 Iranian populations of *Bunium persicum*. Moreover, Ref.^70^ found 10.2 and 11 polymorphic bands among parsley (*Petroselinum crispum* (Mill.) Nym.) samples per ISSR and RAPD primers, respectively. In addition, Mehri et al.^71^ perceived a total of 223 bands, when 69% representing polymorphism among 28 Iranian black cumin genotypes. Agro-morphological characteristics are prerequisites to classify and describe genotype diversity, as stated by Ref.^72^. Our results revealed 25.8% of the appearing polymorphism proportion. This multiplicity was used for measuring the variance among the studied cultivars. Rostami-Ahmadvandi et al.^38^ used 22 ISSR primers for studying genetic diversity between 42 cumin (*Cuminum cyminum*), and 202 bands were produced with polymorphism of 67.32%. 16 unique bands were produced, 4 positive and 12 negative unique bands. Moreover, the dendrogram of our results (Fig. 5) revealed divided the cumin genotypes into two clusters, El Gharbia found in the separated cluster from Assiut, Qena, and El Menia. The second cluster had two sub-clusters, one had El Menia, and the second had two genotypes, Assiut and Qena. Based on supreme genetic distance, clustering of accessions could be helpful in programs of hybrid breeding^33^. Realizing the relationships between environmental factors and genetic diversity presented the effect of environmental conditions on the differentiation and adaptation of cumin accessions. In addition, the genetic diversity of the deliberation accession was assessed with domestic environmental conditions. The relationship between genetic diversity accession wasn’t fully agreed with the ecological conditions of the seed origin^39^. The genetic distances between Assiut and Qena and El Gharbia were 0.12 lower than between El Menia and El Gharbia 0.22 and El Gharbia and Qena 0.2.

On the other hand, salinity can do an inhibition of cell division and growth by inhibiting water uptake and ion toxicity^45,73^. Medicinal plants frequently encounter unfavorable growth conditions due to Ref.^67^. Most of them are temperately tolerant to salinity up to 40 mM NaCl as exposed to fewer germination and germination velocity reduction. However, the diversity in the salt tolerance degree as salinity increased^74^. In our study, significant differences were observed among the cumin landraces at all salinity levels and the interaction between landrace and salinity level was also significant (Figs. 6, 7, 8). An increase in salinity level resulted in decreased germination and seedling development; however, the germination pattern varied among landraces under salt stress and responded differentially to the salinity levels. The above results in Figs. 6, 7 and 8 suggest that seeds of El Gharbia and El Menia landraces can germinate well and rapidly at a lower level of salt (< 6 ds/m), while the decline accelerates with EC of higher than 6 ds/m. Furthermore, the germination and seedling development of Assiut and Qena landraces were ideal in the absence of salt and linearly decreased with salt concentrations increasing. Piri et al.^53^ mentioned that weak cumin seed vigor and seedling development are cumin production problems, followed by increased sensitivity to environmental stresses. This may be explained by the fact that the seed germination decreased in salty conditions due to ion osmotic pressure increased in seed germination and impairment, as well as water absorption reduction by seeds due to high salt level^75^. In addition, seedling growth is a result of cell division and enlargement, while salt stress directly decreases plant development by reducing turgor pressure and assimilate provision, as stated by Ref.^45^. Furthermore, Ref.^76^ mentioned that ion toxicity might reduce important metabolic processes such as dividing and developing cells. Another suggestion was offered by Ref.^50^ which proposed that a photosynthesis disorder happened, resulting in an undesirable effect on the organs of seed production. Some research results on cumin crops^77,78^ suggested that seed germination could be decreased due to the salt stress effect. Our results are approved by the result of the investigation conducted on cumin by Tatari et al.^79^, Vicente et al.^80^ on *Hypericum ericoides,* Han et al.^81^ on adzuki bean cultivar, Ghamarnia et al.^82^ on peppermint, Teimoori et al.^83^ on Camelina, and Fathi et al.^84^ on cotton. As well, Munns et al.^45^ and Morais et al.^73^ reported that the salt stress caused a linear decline in germination and seedling growth characters of all species under study because of osmotic effects; nonetheless, the losses are diverse among the species. However, salt stress stimulates several physiological, biochemical, and morphological responses from plants, depending on the genotype and its development phase^85^. Other reports suggested that different physiological procedures, such as stomatal conductance, osmotic adjustment, photosynthesis, protein synthesis, nucleic acid synthesis, ion uptake, hormonal balance, and enzymatic activity delay plant growth^78,79^. Additionally, it affects the water and ions transport process, resulting in a nutritional imbalance and ion toxicity^80^; accordingly, vegetative growth parameters are harshly affected^86^.

In addition, cumin essential oil was characterized by the existence of monounsaturated, saturated, and polyunsaturated fatty acids. It is well known that fatty acids, the plasma membrane lipid key constituents, play an important role in plant salt tolerance by preserving membrane fluidity, as described by Refs.^87,88^. Figure 1 shows that the fatty acid components of cumin seeds’ essential oil, especially lauric acid, palmitic acid, and linoleic acid, diminished between landraces. In this context, the variations in the ratio of unsaturated and saturated fatty acid constituents are concerned with plant responses to salt stress due to Refs.^83–86^. The correlation coefficient results (Table 5) showed significant differences between different concentrations of salinity for all germination traits. Many reports investigated similar issues and obtained similar results for germination and yield components in different crops, and they found relationships between some characters under different salinity levels^89–91^.

## Materials and methods

The collecting seed samples for the study and protocols were approved by the Horticulture Research Institute’s (HRI) and use committee (ethical approval number: 621329). In addition, the plant collection and use in this research were in accordance with all the relevant guidelines. The current research was conducted at the laboratories in the HRI and Field Crop Research Institute (FCRI), Agricultural Research Center, Giza, Egypt. The present study was divided into four experiments including an assessment of physical characteristics and phytochemical variation in 4 Egyptian cumin landraces, an evaluation of the genetic diversity using ISSR markers, and a study of their sensitivity to salt stress at the germinal stage.

### Plant material

**Figure 9:**
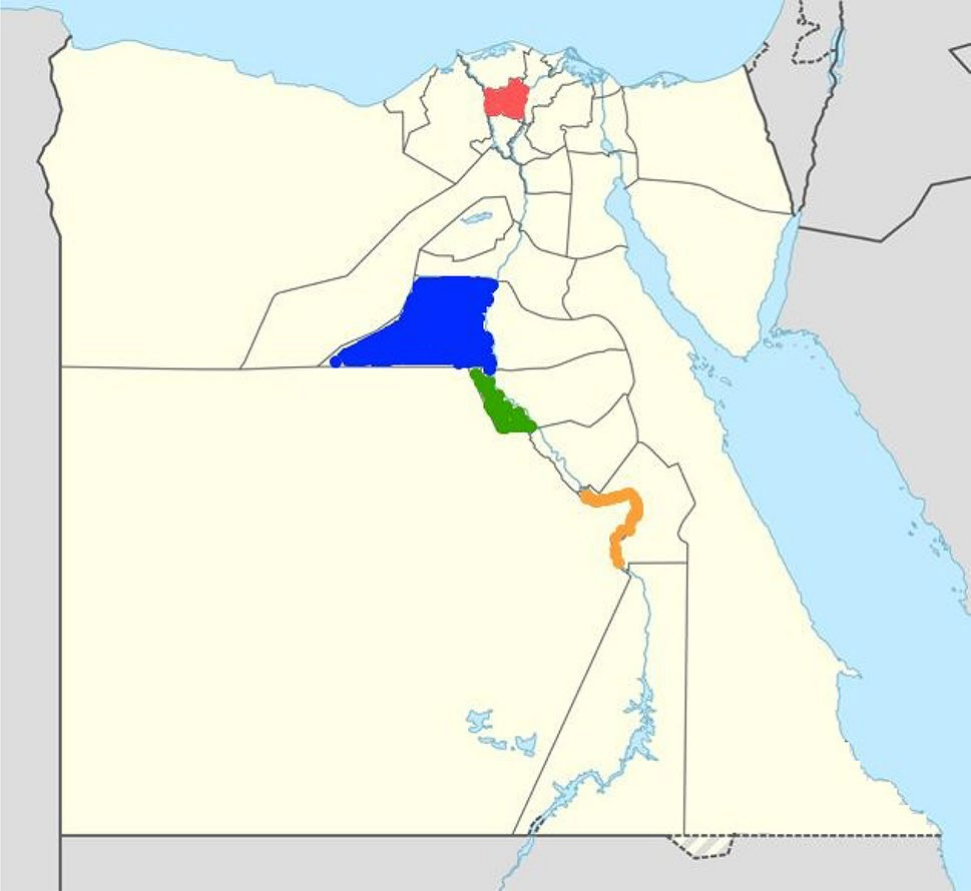
The origins sites of 4 cumin landraces. The sites are El Gharbia (Red); El Menia (Blue); Assiut (Green); and Qena (Orange). The base map of Egypt was downloaded from the web (https://www.geographyknowledge.com/2017/12/egypt-blank-maps.html), and modified by Photoshop software CS5 version, to determine the studied governorates’ location. Last access: 2 June 2023.

**Figure 10:**
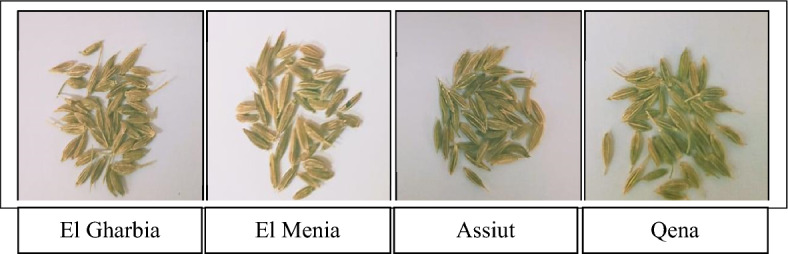
General view of the four cumin landraces.

Under the present investigation, seed samples were collected during the season of 2020/2021 from different geographical areas in Egypt known for growing cumin landraces and leading productive areas. These areas are “El Gharbia, El Menia, Assiut, and Qena”, which represent four Agro-climatic Zones (Fig. 9, Table 6). The climate of all governorates is a hot desert climate. El Menia, Qena, and Assiut have the widest difference in temperatures between days and nights of any city in Egypt; Winters are warm on days but become cool at night. This collection represents the biggest variation for cumin in Egypt. The current laboratory studies were conducted during 2021 and 2022 years at the Agricultural Research Center. The general view of the seeds is shown in Fig. 10.

**Table 6:** Location of the cumin samples studied.

Sites	Province	Lat.	Long.	PET mm day^−1^	Elevation m a.s.l.	*Temperature (°C)		RH (%)
						Max.	Min.	
Nile Delta								
El Gharbia	Qutur	30.97°	30.95°	5.06	14.80	31.26	9.00	63.44
Middle Egypt								
El Menia	Maghagha	28.64°	30.84°	5.74	40.00	31.48	6.74	50.61
Upper Egypt								
Assiut	Dayrout	27.11°	31.06°	5.81	71.00	32.02	6.38	46.18
Qena	Qus	26.10°	32.43°	6.00	72.60	34.79	7.19	36.74

*Source* Climate data were obtained from the agro meteorological unit at Nasa power; https://power.larc.nasa.gov/data-access-viewer/).

*Lat* latitude, *Long* longitude, *PET* potential evapotranspiration, *RH* relative humidity.

*Temperature and relative humidity during the cropping season.

### Physical properties of cumin seeds

#### Seeds dimensions

The seed was placed on a transparent graph paper, and a sample of randomly hundred seeds was selected to examine the average size of the basic dimension “length and width” (mm) of the cumin seed. Measurements were carried out with a digital caliper to an accuracy of 0.01 mm.

#### Mass of 1000 seeds weight

The weight of 1000 seeds (mg) was attained by randomly counting 250 seeds for the desired water content and weighed on an electronic balance with an accuracy of 0.01 g, then multiplied by 4 to get the 1000 seeds’ mass. The reported value was a mean of 3 replications.

#### Seeds bulk density

Seed bulk density is the ratio between the mass and total volume. The determination was made by putting the sample into an empty graduated jar measuring 1000 cm^3^ and measuring its weight^91^. The graduated jar was tapped 10 times in the same manner for all measurements to achieve uniform density in container^92^. It was calculated by the equation of *ρb* = *M*_*s*_/*V*_*o*_, kg m^−3^. Where *ρb* is the bulk density of the sample, (kg m^−3^), *M*_*s*_ is the mass of the sample, kg; *V*_*o*_ represents the volume occupied, m^3,93^.

### Biochemical possessions of cumin seeds

#### Essential oil % and GC–MS analysis

To determine the essential oil (%), the oil was extracted by the hydro-distillation method in a Clevenger apparatus according to the Ref.^94^. As a method described by Guenther^95^, cumin fruits (100 g) were crushed by an electric grinder and immediately placed in 1 l conical flask and connected to the Clevenger apparatus. Five hundred ml of distilled water was added to the flask and heated to the boiling point for hydro distillation. Steam in combination with the essential oils was distilled into a graduated cylinder for an average duration of 3h. The essential oils were collected and dehydrated with sodium sulfate (Na_2_SO_4_) to eliminate traces of moisture and stored in a refrigerator in the dark at 4 °C. The ratio of the amount of oil extracted to the weight of the fruits utilized was used to determine the essential oil percentage.

A 2 µl from the obtained volatile oil was analyzed in the Central Laboratory, Institute of Food Industry and Nutrition, National Research Center, Cairo, Egypt, using gas chromatography (Agilent 8890 GC system) equipped with a mass spectrometer (Agilent 5977B GC/MSD. Samples were diluted with hexane (1:19, v/v). The GC was equipped with an HP-5MS column (30 m × 0.25 μm internal diameter and 0.25 μm film thickness). Analysis was carried out using helium as the carrier gas at a flow rate of 1 ml/min at a split ratio of 20:1, injection volume of 2 µl and the following temperature program: 50 °C for 0 min; rising at 4 °C/min to 240 °C and held for 0 min; rising at 10 °C/min to 280 °C and held for 5 min. The injector and detector were held at 280 °C. Mass spectra were obtained by electron ionization (EI) at 70 eV; using a spectral range of m/z 40–550 and solvent delay of 5 min. Different constituents were identified by comparing the spectrum fragmentation pattern with those stored in Wiley and NIST Mass Spectral Library data.

#### Analysis of fatty acids profile

Fatty acids were determined in the oil using ethyl esters boron tri fluoride method^96^. About 1 ml of 10% NaOH in methanol was added to 100 ml oil. The suspension was heated for 30 min at 85 °C. Petroleum ether was used to extract non-saponifiable lipids, and HCl was then used to saponify them. Fatty acid extract was methylated for 45 min at 60 °C using 1 ml of a 20% methanol solution of boron trifluoride. Then, the extracted fatty acid methyl esters were dissolved in 10 ml heptane for GC analysis. The fatty acid methyl ester (FAME) was quantified by Shematizu Gas Chromatograph Series 2010 equipped with an autosampler (Japan) and interfaced with an FID. The GC was equipped with a temperature-programmable column. The column phase was Supplco DB-Wax (carbowax) with the following dimensions: 30 m long, 0.25 mm i.e. with a 0.25 μm phase thickness. Helium was used as carrier gas with a flow of 40 ml/min. A volume of approximately 1 µl was injected using the inlet in a split mode. The head pressure was set at 2 psi, and the split vent flow was 7 ml/m. The temperature of the injector was 250 °C. At 2 pressure, the column flow rate was 0.68 ml/m. The temperature of the column was held at 260 °C for 80 min after being kept at 200 °C for 10 °C/s. In the selected ion monitoring mode, the detector was functioning. The identification of fatty acids was achieved by obtaining retention times from the FAME standards (Sigma Company, St. Louis, MO). Two GC injections were made for each extraction, as determined. The percentage of total fatty acids was given as the fatty acid content.

### Molecular analysis

The markers of ISSR were used to evaluate genetic diversity in the 4 Egyptian cumin landraces. The DNA was extracted from young leaves of the four cultivars (15-day old seedlings) using the modified CTAB (cetyl trimethyl ammonium bromide, Sigma-Aldrich CAS No. 57-09-0) method according to Kalender et al.^97^ (available at: (http://www.primerdigital.com/DNA)). Ten ISSR primers were used; codes and sequences of the tested primers are shown in Table 7. PCR amplifications were carried out using a Bio-Rad 3.03 version thermocycler. The reaction started with a hot start Taq polymerase at 95 °C for 5 min, and then amplifications were performed for 35 cycles with denaturation at 95 °C for 30 s, annealing temperature (according to ISSR primer) for 30 s, and extension at 72 °C for 90 s. The reaction mixture (20 μl) contained 3 μl sterilized MQ H_2_O, 2.0 μl buffer, 0.4 μl dNTPs, 2.0 μl primer, 1 μl MgCl2, 0.2 μl Taq polymerase, and 0.2 μl template DNA. PCR products were detected on agarose gel (1.2%) in 1× THE buffer (2.4 g Tris-base, 4.76 g HEPES, 1 ml 0.5 M EDTA, dissolved in MQ-water, and brought to the last volume of 100 ml) at a constant voltage of 80 V. Electrophoresis Gene RulerTM DNA ladder solution (Thermo-Scientific—Fermentas, Canada) 500–10,000 base range 25 ng/μl was used. Gels were stained with ethidium bromide (0.5 mg/ml) solution and stored at room temperature. A high-quality gel solution with high sensitivity and resolution using a second-harmonic-generation green laser (FLA—5100 imagine system Fuji photo Film GmbH., Germany) was adopted.

**Table 7:** List of ISSR primers and their sequences and annealing temperature.

Primers	Sequences (5′–3′)	Annealing temperature (°C)
ISSR 810	GAGAGAGAGAGAGAGAT	50
ISSR 835	AGAGAGAGAGAGAGAGYC	55
ISSR 857	ACACACACACACACACYG	55
UBC 808	AGAGAGAGAGAGAGAGC	52
UBC 811	GAGAGAGAGAGAGAGC	51
UBC 814	CTCTCTCTCTCTCTCAT	50
UBC 826	ACACACACACACACACC	52
UBC 827	ACACACACACACACACG	52
UBC 840	GAGAGAGAGAGAGAGATT	52
UBC 901	CACACACACACACACARY	52

### Germination test under salinity stress

To identify the 4 Egyptian cumin landraces tolerant to salt at the germination stage, germination research was performed at the Laboratory of Seed Technology Department, Field Crops Research Institute, Agricultural Research Center. The experiment was laid out in a completely randomized design (CRD) with four replications. Four salinity levels have electrical conductivity “EC” of 4, 8, 12, and 16 ds/m, while the control treatment is considered distilled tap water (0 ds/m). Salinity was artificially created by dissolving the known weight of natural salt crust in distilled tap water. The source of the salt was the salterns of Rashid, El Beheira Governorate, Egypt.

Evaluation of the germination test was performed according to Ref.^98^, whereas 25 seeds of cumin were separately germinated in each replication in sterilized 9-cm diameter Petri dishes covered at the bottom with two sheets of Whitman filter paper. Priority, 10 ml from one respective test solution was poured into the plate. The papers were altered once every 2 days to prevent salt accumulation. Storage conditions and growth of germination stage in the incubator at 25 ± 2 °C, 40% relative humidity, and 16 h day and 8 h night cycle was boarding. The counting of germinated seeds was done every day. A seed was considered germinated when a radicle length of 2 mm was obtained. Landraces’ performance was assessed based on seed germination and seedling growth potential under salinity stress conditions.

### Germination characteristics

Seed Germination (G %): The total number of seeds germinated was counted daily, and the percentage was calculated on the 18th day by the formula of (The number of germinated seeds/The total number) × 100. Germination speed test: Seeds were observed daily for each replicate and considered germinated following radical emergence. Germinated seeds were counted and removed from the Petri dishes. Speed Germination Index (SGI) was calculated as described in the Association of Official Seed Analysis^99^ by the following formula: SGI = n1/d1 + n2/d2 + n3/d3 + ⋯.Where, n = number of germinated seeds, d = number of days. Seeds were considered germinated when the radical was at least 2 mm. Germination Rate (GR) was defined according to the following formula^100^. When, GR = a + (a + b) + (a + b + c)⋯(a + b + c + m)/ n (a + b + c + m). Where a, b, and c are No. of seedlings in the first, second, and third count, m is no. of seedlings in the final count, and n is the number of counts. Mean Germination Time (MGT): Mean Germination Time (MGT) was calculated based on the following equation^101^. MGT = Σ Dn/Σn, where (n) is the number of seeds, that were germinated on the day, and D is the number of days counted from the beginning of germination. Germination Index (GI): ∑ Gt/T, where Gt is the number of seeds germinated on day t, and T is the number of days after sowing^102,103^. Germination Vigor Index (GVI): ∑Gt/T × AFW, where AFW is the mean seedling fresh weight.

### Seedling characteristics

Seedling root and shoot length (cm): ten normal seedlings 18 days after planting were measured. Seedling fresh and dry weight (g): Ten normal seedlings 18th days after planting were measured to determine the fresh weight, then the seedlings were dried in a hot-air oven at 85 °C for 12 h to obtain the seedlings’ dry weight (g). Seedling Water Content (WC%): was determined on the 18th day from planting, according to the formula WC (%) = (FW − DW/FW) × 100, where FW = fresh weight and DW = dry weight (Black and Pritchard, 2002). Seedling vigor (SV): Seedling vigor was determined as the product of the germination percentage and that of seedling length and seedling dry weight. It was calculated according to Ref.^104^ as Vigour index I = Germination (%) × Seedling length (Root + Shoot). Vigour index II = Germination (%)× Seedling dry weight (Root + Shoot).

### Phenotypic variability in cumin landraces

Pooled data were used to estimate the simple phenotypic correlation coefficient between all possible pairs of the studied traits according to Ref.^105^ for landraces grown under the different salinity levels of EC; control, 4, 8, 12, and 16 ds/m.

### Data analysis

Levene’s test was used to examine the homogeneity of the variance to analyze data by SPSS program (version 29.0). One-way ANOVA was conducted for the experiment. Duncan test (α = 5%) was used to compare means. For the ISSR marker, the band profiles were rated as absent (0) or present (1) for distinct, reproducible bands. ISSR primers’ banding patterns were measured to determine the degree of genetic relatedness between the samples under investigation. The genetic data was analyzed using binary values (1, 0), and the results were utilized to generate a phenogram that deals with the genetic links among the genotypes investigated. The genetic data were analyzed using MEGA5.1.; Molecular Evolutionary Genetics Analysis, version 7^106^, (available at: http://www.megasoftware.net/). The method applied is based on cluster analysis expressing the relationships of the studied cultivars as distance percent in a cluster tree and similarity matrix. The primer is analyzed according to, heatmap using R software available at https://irscope.shinyapps.io/iMEC/^107^, and PCA (principle component analysis) using PAST software, available at https://biit.cs.ut.ee/clustvis/ according to Ref.^108^.

### Consent to participate

All authors agreed to participate in the present work.

## Conclusion

This research discovered that geographical location and genetics have a major impact on the phenotypic and chemotype of cumin landraces. Assuit landrace produced the highest essential oil percentage (8.04%), whereas Qena landrace produced the most cumin aldehyde (25.19%); the main substance in essential oil. Furthermore, El Gharbia landraces gave the maximum lauric acid (62.73%) as the primary component in fatty acid. The genetic diversity of the different cumin landraces was analyzed using ISSR markers for the first time in Egypt. Results showed a relatively high ISSR markers efficiency for discriminating among the various landraces. Furthermore, reasonably high differentiation and gene flow were detected among the geographical sites. In addition, the results demonstrated that the cumin tolerance to the salt significantly differs between landraces. El Gharbia and El Menia seeds showed a moderate tolerance to salinity at 4 ds/m. While Assuit and Qena seeds represent sensitivity to salt stress. In addition, Qena landraces showed supreme germination and seedling values among other landraces under 12 and 16 ds/m. Moreover, significant differences between the different salinity concentrations for all germination traits. According to the findings of this study, breeders might employ these characteristics in cumin selection and breeding programs to generate desirable cultivars.

### Supplementary Information

Supplementary Information.

## Data Availability

All data generated or analyzed during this study are included in the article and its Supplementary Information.
