# Supplementary material for: Comparative studies of four cumin landraces grown in Egypt
Source: Sci Rep. 2024 Apr 5;14:7990. doi: 10.1038/s41598-024-57637-3 (PMC10997781; doi:10.1038/s41598-024-57637-3)
Supplement: Supplementary file 1 — Supplementary Information. [file 41598_2024_57637_MOESM1_ESM.docx]

**Supplementary Data**

The gel images have been modified before depending on your request before. The original images were captured in one image, and the samples were arranged and injected into the gel as in the first image, purely meaning that the three primers are in one image with one marker.

Then when you requested to make an edit and split the images, the images were cut and the marker was transferred to each primer to separate from each other.

**Here below are the original blots images**


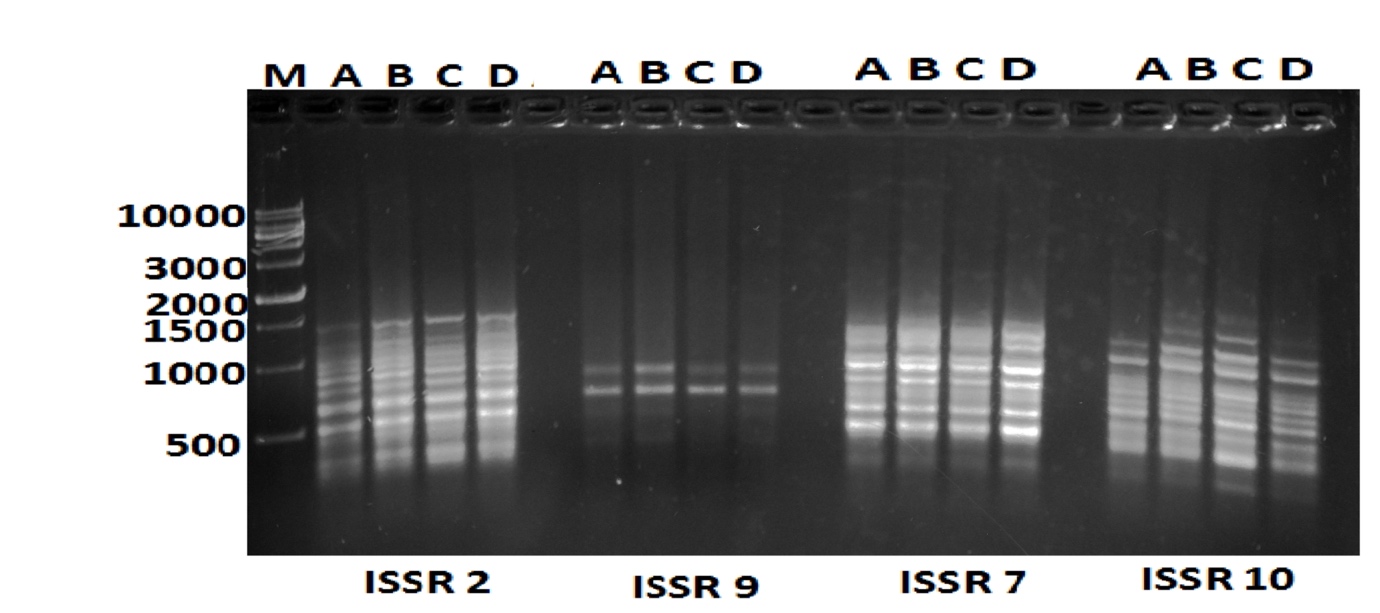


**
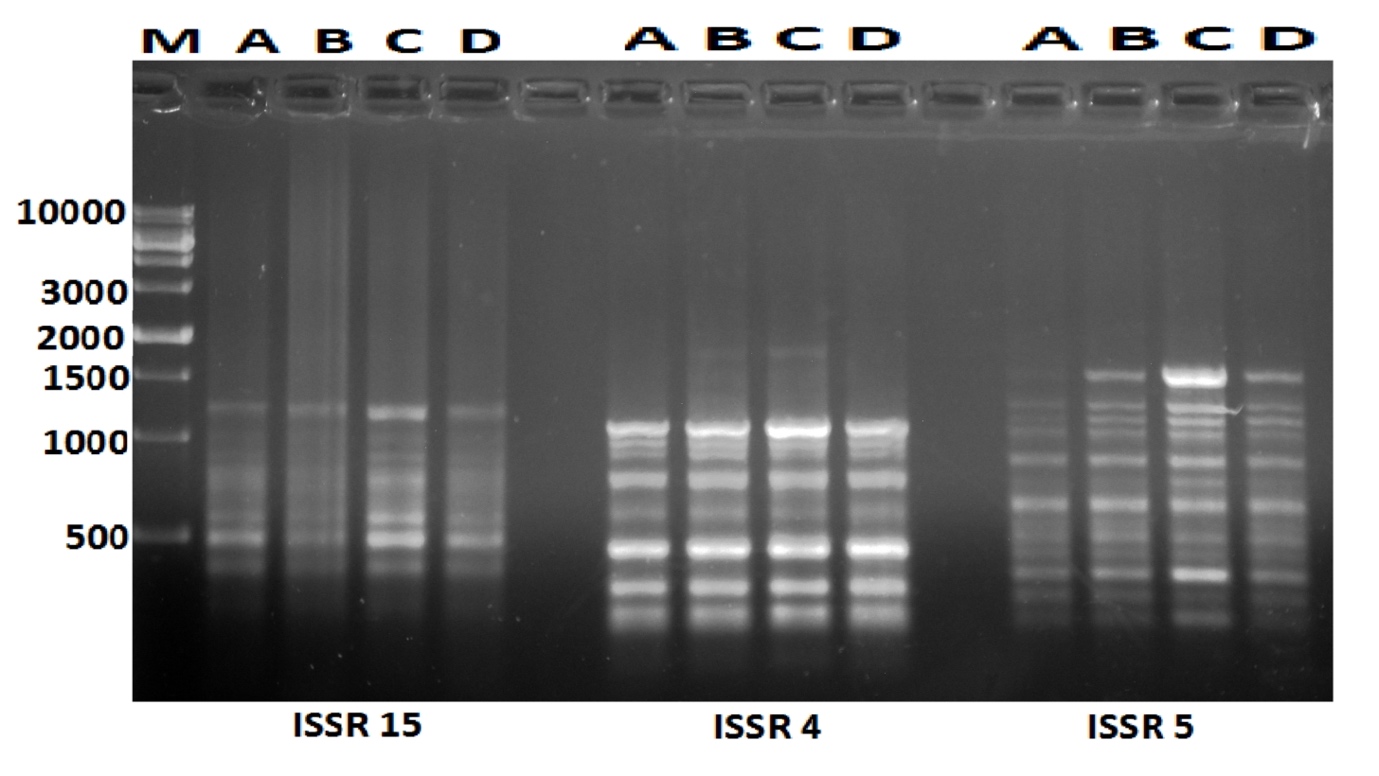
**

**
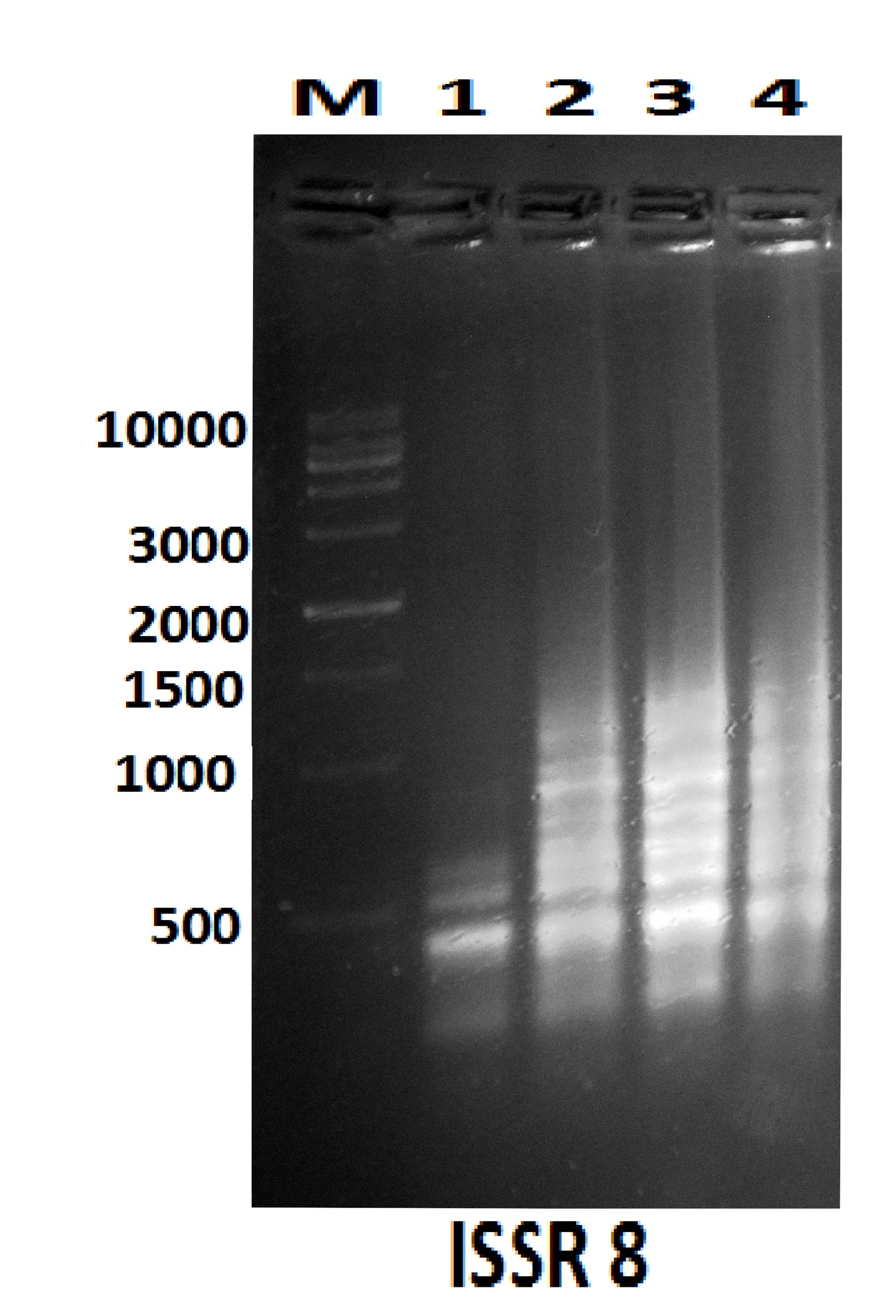

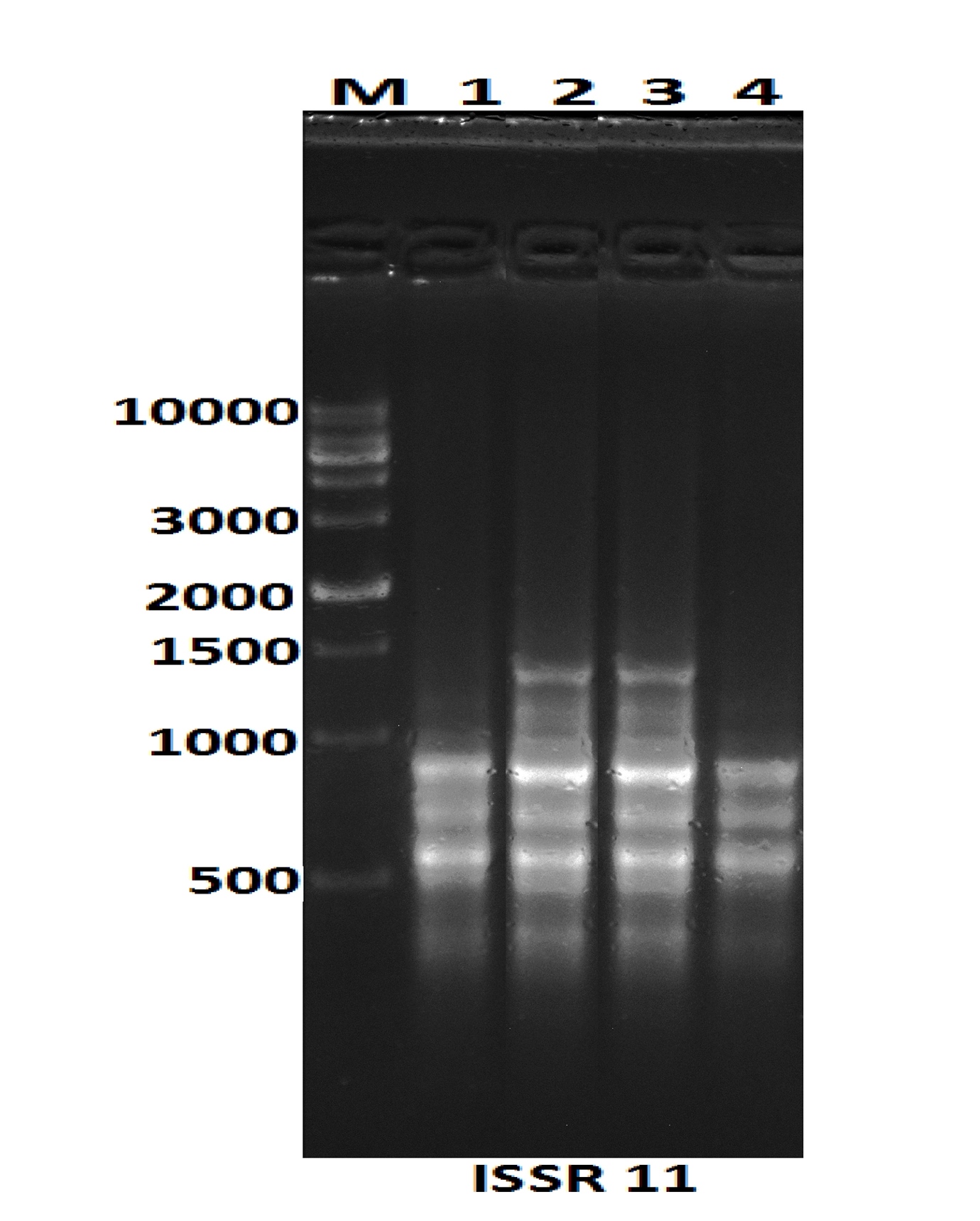

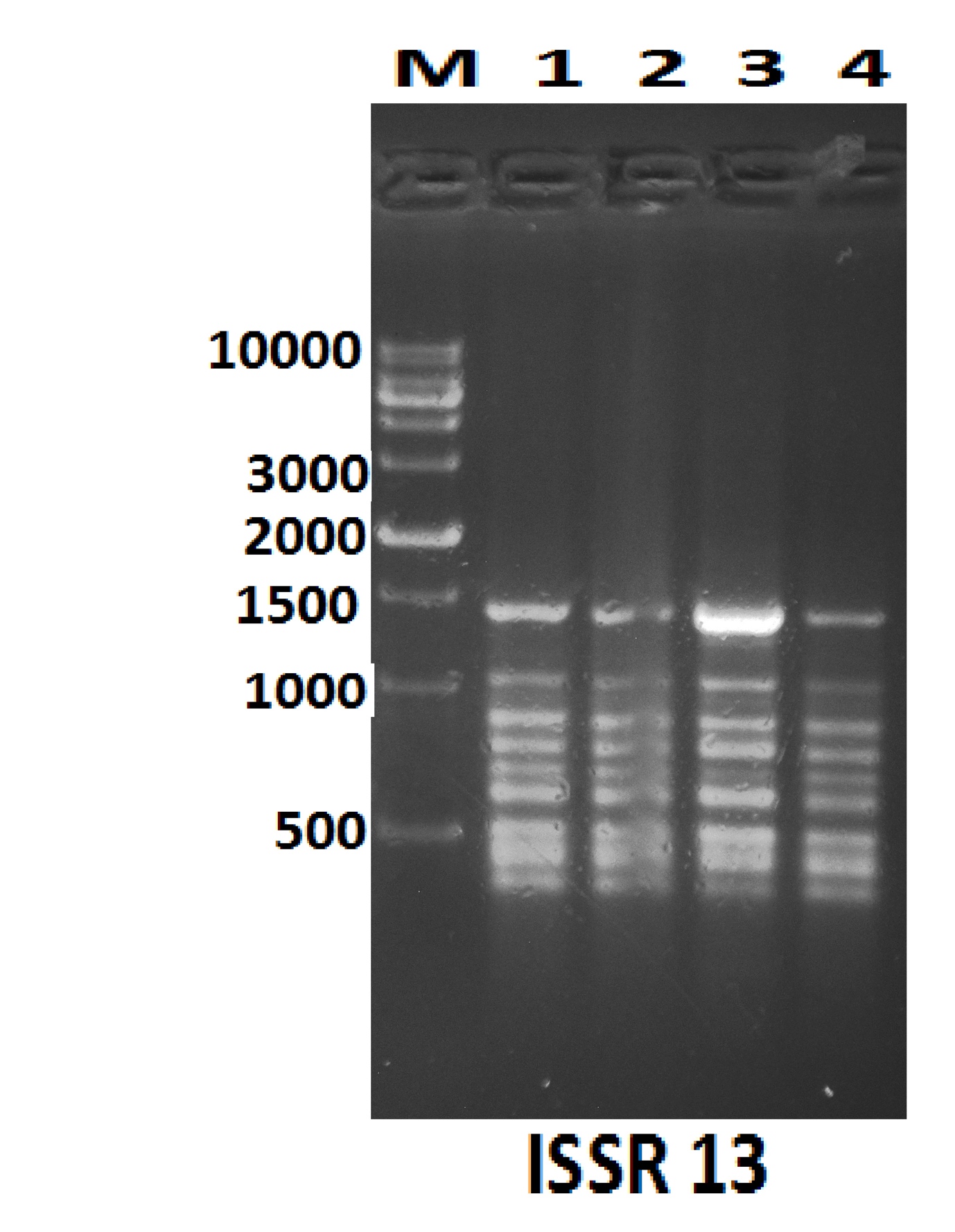
**
